# Measuring and analyzing the emotional engagement exhibited by chinese university students in ideological and political theory courses via the IPTCSSE

**DOI:** 10.1371/journal.pone.0341669

**Published:** 2026-02-10

**Authors:** Liangliang Wang

**Affiliations:** School of Marxism, Xi’an Shiyou University, Xi’an, China; Xi'an University of Posts and Telecommunications, CHINA

## Abstract

This study investigates the emotional responses and engagement levels of Chinese university students enrolled in Ideological and Political Theory Courses (IPTCs), with particular attention to how curriculum design influences students’ attitudes and feelings. Using the IPTCSSE scale, a quantitative analysis was conducted to measure multiple dimensions of emotional engagement. A total of 3,992 valid questionnaires were collected. Based on the emotional engagement subscale, exploratory and confirmatory factor analyses identified seven underlying factors: interest and belonging, classmates and peers, family and curriculum, emotions and feelings, sense of identification, teacher support and influence, and sense of achievement in courses. Descriptive statistics indicated that students generally evaluated all seven factors positively, and all factors were significantly and positively correlated. Among them, sense of achievement in courses received the highest score, while classmates and peers scored the lowest. Correlation and linear regression analyses further showed that each factor positively predicted emotional engagement in IPTCs. Interest and belonging emerged as the strongest predictor, whereas sense of achievement exerted the smallest predictive effect. Drawing on these findings, the study discusses how emotional engagement can be strategically emphasized to improve the pedagogical effectiveness of IPTCs. The results highlight the importance of fostering interest, belonging, supportive teacher-student relationships, and positive emotional experiences to enhance students’ overall engagement with ideological and political education in China’ s higher education context.

## 1. Introduction

Student engagement has long been recognized as a key indicator of teaching effectiveness and learning quality in higher education. In recent years, scholars have increasingly emphasized that engagement is not a unidimensional construct, but a multifaceted phenomenon encompassing behavioral, cognitive, and emotional dimensions [1].Among these, emotional engagement—students’ affective reactions toward learning activities, teachers, and content—plays a particularly important role in shaping motivation, persistence, and academic achievement [2]. Despite its significance, emotional engagement has received comparatively limited empirical attention, especially within value-oriented and ideologically charged educational settings such as ideological and political theory courses (IPTCs) in China.

In the context of Chinese higher education, IPTCs are foundational courses designed to cultivate students’ Marxist worldview, socialist values, and moral integrity. While these courses carry strong political and moral expectations, they often face challenges in stimulating students’ active participation and emotional resonance. Prior research has largely concentrated on students’ ideological identification, cognitive understanding, or behavioral participation, whereas the affective dimension of engagement, the extent to which students experience emotional involvement, interest, or empathy toward ideological content—remains underexplored. As a result, a few is known about how students’ emotional engagement influences their overall learning experience and ideological internalization.

To address this gap, it is essential first to define the key construct that underpins this study. Drawing upon Fredricks et al. (2004), this research conceptualizes emotional engagement as students’ affective investment in learning, reflected in their feelings of enthusiasm, enjoyment, interest, and attachment toward course content and instruction [3].From the perspective of Pekrun’s (2006) control–value theory, such emotions are shaped by students’ perceived control over learning outcomes and the value they assign to learning tasks [4].In IPTCs, emotional engagement thus reflects students’ willingness to emotionally identify with socialist ideals and to connect personal aspirations with collective values—a process central to the success of ideological education.

Given the pivotal role of emotional engagement in linking cognition and value formation, understanding its characteristics and influencing factors in IPTCs is both theoretically and practically significant. However, the existing literature offers few empirically validated tools for assessing emotional engagement in this specific context. To fill this gap, the present study employs the Ideological and Political Theory Course Student Engagement Scale (IPTCSSE) to measure and analyze the emotional engagement of Chinese university students,

By doing so, it aims to answer the following research questions: (1) What is the overall level of emotional engagement exhibited by Chinese university students in IPTCs? and (2) What factors significantly influence this engagement? The findings are expected to enrich the theoretical understanding of engagement and offer pedagogical insights for enhancing the effectiveness of ideological and political education in higher education.

The study was designed to address two primary objectives. First, to understand the emotional responses and engagement levels of students in Ideological and Political Theory Courses (IPTCs). To examine how the curriculum influences students’ attitudes and feelings toward ideological and political education. Second, to achieve the first objective, we employed the Ideological and Political Theory Course Student Engagement Scale (IPTCSSE). This validated instrument measures three dimensions of engagement—behavioral, cognitive, and emotional. The emotional engagement dimension specifically captures student affective responses, including interest, enthusiasm, enjoyment, and identification with courses’ content. Quantitative analysis of Likert-scale responses allows us to assess engagement levels systematically across the sample.To address the second objective, we included qualitative reflection items within the questionnaire. Students were asked to describe specific experiences or curriculum elements that elicited emotional responses or influenced their attitudes. These open-ended responses provide rich insights into how the curriculum shapes students’ feelings, enabling a nuanced understanding of the relationship between instructional practices and emotional engagement.

By integrating quantitative and qualitative methods, the study ensures methodological alignment with the research objectives. Quantitative measures allow for reliable assessment of engagement levels, while qualitative reflections provide contextual understanding of the curriculum’s impact. This mixed-method design facilitates triangulation, strengthening the validity of conclusions regarding students’ emotional engagement and the curricular factors influencing it.

## 2. Literature review

### 2.1. Conceptualizing student engagement

The concept of student engagement has evolved into a central construct in contemporary educational research, representing students’ overall involvement in learning activities at cognitive, behavioral, and emotional levels. According to Fredricks, Blumenfeld, and Paris (2004), engagement is a multidimensional construct that unites the energy and effort students bring to their academic experiences.This tripartite model—comprising behavioral, cognitive, and emotional dimensions—has become the dominant framework in engagement studies. Behavioral engagement refers to participation in academic and social activities; cognitive engagement involves investment in learning and self-regulated strategies; and emotional engagement encompasses students’ affective reactions to teachers, peers, and learning content, such as interest, enthusiasm, pride, or boredom.

Subsequent scholars, including Kahu (2013) [5]and Trowler (2010) [6]extended this understanding by linking engagement to broader socio-cultural and institutional contexts, emphasizing that emotional engagement serves as the affective bridge between individual motivation and institutional learning outcomes.Emotional engagement is particularly critical in courses where personal values, moral reasoning, or ideological understanding are core learning outcomes. Unlike cognitive engagement, which reflects how students think about learning, emotional engagement reveals how students feel about learning—a distinction especially relevant to ideological education.

### 2.2. Emotional engagement and its educational significance

Emotional engagement has been recognized as both a determinant and consequence of effective learning. Grounded in Pekrun’s (2006) Control-Value Theory of Achievement Emotions, emotional engagement is shaped by two psychological appraisals: perceived control over learning and the value students attach to academic tasks. Positive emotions such as enjoyment, pride, and interest emerge when students perceive high control and value; conversely, low control or low value often leads to boredom or anxiety. These emotions, in turn, influence learning motivation, persistence, and performance [7].

Here, we should distinguish between Emotional Engagement(EE) and Affective Engagement(AE). Emotional engagement and affective engagement are related but distinct concepts in psychology and education. Several core differences distinguish them. First, the nature of EE refers to specific, intense emotional responses—such as joy, frustration, or anxiety—while AE involves a broader emotional connection and attitude toward an activity. Second, emotional engagement tends to be momentary and fluctuating, whereas affective engagement is more stable and enduring. Third, EE focuses on reactions to specific events or stimuli, while AE reflects an overall emotional disposition and sense of value. Fourth,EE can be highly intense, whereas AE is generally milder yet persistent. For example, feeling excited after solving a difficult problem represents EE, whereas consistently enjoying math class reflects AE.

In practical terms, EE describes the immediate emotional reactions students experience in the moment—such as feeling proud upon receiving praise or frustrated when facing a challenge. AE, on the other hand, refers to a student’s ongoing emotional relationship with an activity, encompassing whether they generally like or dislike the subject, feel connected to it, and find it meaningful over time. One might compare emotional engagement to daily weather, which changes frequently, while affective engagement resembles the climate, representing a long-term pattern.

Despite these differences would be exist conceptually, in the context of IPTC teaching discussed in this article, we treat the two concepts as equivalent for two main reasons. First, affective/emotional engagement is defined here as affiliation with and identification with the university—including its staff and students—as well as the emotions experienced during academic tasks. Second, whether termed emotional or affective engagement, both refer to students’ emotional responses to teachers, peers, learning, and school. From this perspective, engagement is seen as fostering connections with the university and influencing students’ willingness to apply themselves to their studies [3]. In higher education, emotional engagement has been associated with improved learning satisfaction, deeper cognitive processing, and stronger commitment to course goals [8]. When students experience positive emotions toward learning, they are more likely to participate actively, internalize content meaningfully, and sustain motivation in the face of challenges. Conversely, disengagement or emotional detachment can lead to superficial learning and decreased academic achievement.

Despite its importance, empirical research on emotional engagement remains limited compared to the behavioral and cognitive dimensions. Many studies treat engagement as a single construct or focus primarily on observable behaviors such as attendance or participation. As a result, the emotional aspects of student engagement—how feelings of interest, empathy, or identification influence learning—have been under-theorized and under-measured. This gap is particularly salient in disciplines where emotional resonance with course values is essential, such as ideological and political education.

### 2.3. Engagement in Ideological and Political Theory Courses (IPTCs)

In the Chinese higher education system, Ideological and Political Theory Courses (IPTCs) are designed to cultivate students’ political literacy, value consciousness, and moral responsibility in accordance with socialist ideals. These courses aim not only to transmit theoretical knowledge of Marxism and Chinese political thought but also to foster emotional identification with national development goals and collective values. However, achieving this dual objective—intellectual understanding and emotional resonance—has proven challenging. Existing studies on IPTCs often highlight issues of student passivity, limited classroom participation, and perceived irrelevance of content to personal life [9]. Research tends to concentrate on improving teaching methods, such as case-based learning, multimedia integration, or ideological storytelling, to enhance behavioral participation and cognitive comprehension. While these efforts contribute to pedagogical innovation, few studies explicitly examine the emotional dimension of student engagement—how students’ affective experiences influence their learning outcomes and ideological identity formation.

Moreover, measurement in this field remains largely qualitative or descriptive. Few empirical tools have been developed to assess emotional engagement systematically in IPTCs. The absence of validated instruments limits comparative analysis across universities and regions. The recently developed Ideological and Political Theory Course Student Engagement Scale (IPTCSSE) represents an important step forward, as it enables quantitative evaluation of students’ emotional, cognitive, and behavioral engagement in these courses. Nonetheless, empirical studies applying this framework remain scarce, particularly in relation to identifying the factors that shape emotional engagement among Chinese university students.

Thus, it is vital that most Chinese university students have engaged emotionally with ideological and political theory courses (IPTCs), especially in recent years when socialism with Chinese characteristics preceded a new era. This research aims to examine the emotional responses and engagement levels of students in these courses to understand how the curriculum impacts their attitudes and feelings. This is particularly relevant in the context of educational reforms and the evolving landscape of political education in China.

### 2.4. Factors influencing emotional engagement

Scholars have identified multiple interrelated factors that affect students’ emotional engagement in higher education. These can be categorized into teacher-related, course-related, environmental, and student-related factors.

#### 2.4.1. Teacher-related factors.

Teachers play a central role in shaping students’ emotional experiences. Research consistently shows that teacher enthusiasm, empathy, and emotional expressiveness can enhance students’ affective connection to learning [10]. In IPTCs, where teachers serve as both knowledge transmitters and ideological guides, their emotional charisma and ability to communicate values persuasively are critical. Studies have found that teachers who use storytelling, real-life examples, and emotionally charged narratives foster stronger emotional identification and ideological resonance among students [11]. Conversely, an overly didactic or authoritative teaching style can suppress emotional engagement and generate resistance.

#### 2.4.2. Course-related factors.

The design and content of the course also significantly influence emotional engagement. Learning materials that are relatable, contextually relevant, and contemporary tend to evoke greater emotional responses. The integration of multimedia, digital platforms, and interactive activities has been shown to increase students’ sense of immersion and enjoyment [12]. Moreover, opportunities for discussion, reflection, and creative expression enable students to connect abstract ideological concepts with personal experiences, thereby deepening emotional resonance. A rigid curriculum that prioritizes memorization over meaning-making, by contrast, often limits students’ affective investment.

#### 2.4.3. Environmental and contextual factors.

Emotional engagement is also affected by the broader learning environment, including classroom climate, institutional culture, and peer interactions. A supportive atmosphere that encourages open discussion and critical thinking enhances students’ sense of belonging and psychological safety, which are prerequisites for emotional involvement [13]. In the context of IPTCs, where topics can be politically sensitive, creating a respectful and dialogic classroom climate is essential. The institutional emphasis on ideological conformity can sometimes discourage emotional authenticity, thereby moderating students’ affective responses.

#### 2.4.4. Student-related factors.

Individual differences such as motivation, prior ideological beliefs, emotional regulation ability, and academic self-efficacy also play crucial roles. Students who perceive ideological learning as personally meaningful are more likely to experience positive emotions such as pride or inspiration. Conversely, those who view it as externally imposed may experience apathy or resistance. Furthermore, generational differences in media literacy and information exposure affect how students emotionally process ideological narratives. Younger students who are accustomed to digital and multimodal communication may engage more readily with innovative pedagogies that combine ideological content with new media storytelling [14].

In sum, emotional engagement arises from the dynamic interaction of personal, pedagogical, and contextual variables. Understanding these factors holistically is essential for enhancing teaching effectiveness and promoting genuine value internalization in IPTCs.

### 2.5. Theoretical implications and practical background

Many researchers have studied the theoretical aspects of emotional engagement. Connell et al. (1991) suggested that emotional engagement, such as interest, joy, fatigue, or anxiety, is typically reflected in students’ emotional responses in the classroom [15]. Lee and Smith (1995)assessed emotional engagement by measuring students’ emotional responses to schools and teachers [16]. Voelkl (1997) conceptualized it as identification with the school [17], whereas Finn (1989) defined it as a sense of belonging (viewing school as important) and value (appreciation for school-related outcomes) [18]. Kim, Lee, Song, & Lee (2021) noted that persistent emotional engagement includes students’ interest in and enthusiasm for school and reflects students’ reactions in the classroom as well as their feelings about achieving academic success [19]. Students who excel academically often experience satisfaction and pride, whereas those who struggle may lack these positive emotions [20]. High emotional engagement typically correlates with high cognitive engagement, whereas low emotional engagement is associated with reduced cognitive involvement [8]. Additionally, emotional engagement is closely tied to academic performance and dropout rates [21], and it influences learners’ emotional responses [22], which can range from enjoyment and interest to boredom and dissatisfaction. If a course fosters happiness, joy, and deep interest, students’ emotional engagement is likely to increase [23].

From a theoretical standpoint, integrating Fredricks et al.’ s (2004) multidimensional engagement model with Pekrun’ s (2006) control-value theory offers a robust analytical foundation. It allows emotional engagement to be understood both as an outcome—reflecting how students feel in response to learning experiences—and as a mediating mechanism that connects instructional practices to ideological identification. This theoretical integration highlights the importance of measuring emotional engagement empirically to uncover the pathways through which pedagogical and contextual factors influence students’ ideological and moral learning outcomes.

When teachers set specific emotional goals in the classroom, some students may engage with these goals primarily to gain rewards or credits, but their engagement may not necessarily reflect their genuine attitudes and beliefs about the subject [24]. Teachers’ emotional support influences learners’ basic psychological needs, emotions, and emotional engagement. Online training in management education supports emotional engagement and problem-solving skills [25]. Even mobile learning affects student engagement [26]. The relationships between the emotions and achievements of EFL learners in China have been examined, and engagement was found to be a mediator [27].Researchers often separate the subjective and emotional aspects of the affective domain from the more rational, goal-oriented cognitive domain. In classrooms, course content is typically viewed as objective and distinct from attitudes, values, beliefs, and emotions [28].Due to the perceived difficulty in measuring affective outcomes compared to cognitive outcomes, education systems commonly prioritize cognitive assessments [29]However, Ben-Eliyahu et al. (2024) argued that emotional engagement can significantly influence the learning process, as seen in characteristics such as isolation, rebellion, compliance, innovation, involvement, and adherence to norms [30]. Previous studies have shown that positive learning emotions can enhance academic performance [31].

### 2.5. The rationale and research gap

The rationale for this research can be outlined as follows. First, in recent years, Chinese universities have increasingly focused on ideological and political education as part of their curriculum reforms [32]. Understanding how students engage emotionally with these courses is crucial for assessing the effectiveness of this educational strategy and ensuring that they meet their intended goals [33–35]. Moreover, emotional engagement is a key component of effective learning. By measuring students’ emotional responses to IPTCs, this research aims to gain insight into how these courses influence students’ attitudes and involvement. Emotional engagement can impact learning outcomes, retention, and the overall educational experience [36]. The Ideological and Political Theory Course Student Emotional Engagement (IPTCSSE) scale, originally adopted from the National Survey of Student Engagement (NSSE), is employed to provide a structured and validated approach to measuring emotional engagement. This tool allows for a nuanced analysis of students’ emotional responses and provides a clear picture of their engagement level [37]. Furthermore, this research contributes to the literature by focusing specifically on the emotional aspects of engagement in IPTCs, an area that has not been studied extensively. It aims to fill gaps in the understanding of how these courses are received by students and how their emotional responses can inform improvements in course design and delivery. Finally, the findings of this study have practical implications for educators and policymakers. By identifying factors that enhance or hinder emotional engagement, this research can provide recommendations to improve course content, teaching methods, and student support strategies to foster more effective learning environments in ideological and political education. Overall, this research aims to offer a comprehensive analysis of emotional engagement in a critical area of education with the goal of enhancing the effectiveness and impact of IPTCs at Chinese universities.

Although prior studies have examined various aspects of engagement and teaching innovation in IPTCs, several research gaps remain. First, the emotional dimension of engagement has not received sufficient empirical attention. Most existing studies focus on cognitive comprehension or behavioral participation, overlooking how affective experiences shape students’ ideological development. Second, there is a lack of validated measurement tools specifically tailored to the ideological and political education context. The IPTCSSE provides a new opportunity to quantify emotional engagement, but empirical applications are still limited. Third, while previous research has discussed teaching reform strategies, few studies have systematically analyzed the factors influencing emotional engagement through a theoretical lens such as the control-value framework.

### 2.7. Summary

The research assesses how emotionally engaged Chinese university students are when they participate in IPTCs. The study uses surveys to measure various aspects of emotional engagement, such as interest, enthusiasm, and emotional responses, during these courses. The research also evaluates how the content of these courses influences students’ emotional engagement and whether students feel a sense of relevance, connection, and motivation in IPTCs. Various demographic factors (e.g., gender, major) that affect emotional engagement are analyzed to help determine whether certain groups are more or less engaged and why. We explore how different teaching methods (e.g., traditional lectures vs. interactive discussions or multimedia presentations) impact emotional engagement. Finally, this research tracks changes in emotional engagement over the course of a semester or academic year to provide insight into how engagement evolves with increased exposure to the material. Based on the findings, this study suggests recommendations for improving the effectiveness of IPTCs by advocating for more engaging or relevant content and teaching approaches to enhance student engagement. The results provide valuable insights into how IPTCs can be tailored to better resonate with students to foster a more engaging and impactful learning experience.

In summary, the existing literature underscores the significance of emotional engagement as a central yet underexplored component of student learning, particularly within the context of ideological and political education. Prior research has not fully addressed how teacher characteristics, course design, learning environments, and student factors interact to influence emotional engagement in IPTCs. Moreover, there remains a lack of quantitative evidence and theoretical integration in this field. Addressing these gaps, the present study employs the Ideological and Political Theory Course Student Engagement Scale (IPTCSSE) to measure and analyze emotional engagement among Chinese university students. By identifying its levels and key influencing factors, this research seeks to contribute to both the theoretical enrichment of engagement studies and the practical enhancement of ideological and political theory teaching in higher education.

## 3. Methods

### 3.1. Survey design

The main instrument used in this study was a structured questionnaire designed to measure the multidimensional construct of student engagement in Ideological and Political Theory Courses (IPTCs). The questionnaire was adapted from the Ideological and Political Theory Course Student Engagement Scale (IPTCSSE), originally developed based on Fredricks et al.’s (2004) tripartite model of engagement and subsequent theoretical refinements by Reeve and Tseng (2011) [38] and Kahu (2013).The IPTCSSE has been validated in several studies involving Chinese university students and is particularly suited to capturing engagement in the affective, cognitive, and behavioral domains of ideological and political education. Minor modifications were made to ensure linguistic clarity, contextual relevance, and alignment with the research objectives of the present study.

Section two consisted of the student engagement scale, which contained 45 items across three subdimensions. Behavioral engagement (15 items) measured students’ observable participation and effort during class (e.g.,‘I actively participate in classroom discussions’).Cognitive engagement (15 items) assessed students’ investment in understanding course content and applying it to real-life contexts (e.g.,‘I try to connect theoretical concepts from the course with current social issues’).Emotional engagement (15 items) captured students’ affective responses to learning experiences, including enthusiasm, enjoyment, empathy, and emotional identification with socialist values (e.g., ‘I feel inspired by the examples used in the course’ and ‘I am proud to learn about the achievements of socialism with Chinese characteristics’).All items in Section Two were measured using a ten-point Likert scale, ranging from 1 (‘strongly disagree’) to 10 (‘strongly agree’). Higher scores indicated stronger engagement in the corresponding dimension. The internal consistency reliability (Cronbach’s α) for the overall scale and each subdimension exceeded 0.85, suggesting satisfactory internal reliability. Section Three included two open-ended reflective questions designed to elicit richer qualitative data on students’ emotional experiences in IPTCs. Students were invited to describe (a) specific moments in which they felt emotionally connected or disconnected from the course and (b) their views on what aspects of teaching most influenced these emotional reactions. These qualitative responses provided complementary insights that could not be fully captured by numerical ratings, thereby enriching the interpretation of quantitative findings.

The final questionnaire comprised four main sections. Section one collected demographic information and background variables that may influence engagement, including gender, academic year, major, and previous exposure to ideological and political education. These data were used for descriptive analysis and potential covariate control in subsequent statistical tests.

The decision to combine quantitative and qualitative measurement methods was guided by both theoretical and methodological considerations. Emotional engagement, as emphasized in Pekrun’s (2006) control-value theory, involves complex affective appraisals that are not always reducible to Likert-scale scores. Open-ended responses therefore served as a means of methodological triangulation, enhancing construct validity and ensuring that students’ subjective meanings and emotional nuances were adequately represented [39]. The integration of these methods also aligns with the exploratory nature of the present study, which seeks not only to measure emotional engagement levels but also to understand the contextual factors that shape them.

In summary, the adapted questionnaire combines established theoretical foundations with contextual sensitivity to Chinese ideological and political education. The use of mixed methods enables a more comprehensive assessment of students’ emotional engagement—both as a measurable construct and as a lived emotional experience—thus providing a robust empirical basis for addressing the research questions of this study.

### 3.2. Sampling

A representative sample of college students from various Chinese universities was selected. Diversity was ensured in terms of major, year of study, and demographic characteristics. Upon inputting the data into the statistical software, 123 noncompliant questionnaires were identified through high-low grouping, leaving 3992 questionnaires for further analysis. These 3992 questionnaires constituted the final sample for the data analysis. The frequency statistics revealed the composition of the groups included in this analysis.

### 3.3. Data collection

The questionnaire was distributed to the selected sample. Paper surveys were used to ensure high response rates and reliable data for the data analysis. Data were collected from undergraduate students enrolled in Ideological and Political Theory Courses (IPTCs) at three Chinese universities during the spring and autumn semesters. The questionnaire was distributed to the selected sample using a mixed method: paper-based questionnaires were administered in-class during IPTC lectures, and an online version was shared via the universities’ official learning management systems for students who could not attend in person. This approach ensured comprehensive coverage of the selected sample while allowing students to complete the survey at their convenience.

Participation was voluntary, and all respondents provided informed consent prior to completing the questionnaire. Students were assured of the confidentiality of their responses and their right to withdraw from the study at any time. A total of 3992 valid responses were collected, yielding a valid response rate of 97.01%. To provide a clear overview of participants’ backgrounds, key demographic characteristics are summarized in Table 1.

**Table 1:** Demographic characteristics of participants (N = 3992).

Variable	Category	Frequency (n)	Percentage (%)
Gender	Male	1764	44.2
	Female	2228	55.8
Academic Year	First year	1142	28.6
	Second year	1329	33.3
	Third year	1005	25.2
	Fourth year	516	12.9
Major	Humanities & Social Sciences	1628	40.8
	Science & Engineering	1664	41.7
	Economics & Management	700	17.5
Prior IPTC Experience	Yes	2858	71.6
	No	1134	28.4
Age Range	18–20 years	2191	54.9
	21–22 years	1385	34.7
	23 and above	416	10.4

As shown in Table 1, female students slightly outnumbered male students (55.8% vs. 44.2%). The sample was fairly evenly distributed across academic years, with the largest proportion in the second year (33.3%). Most participants were majoring in either humanities, social sciences, or science and engineering disciplines. Approximately 71.6% of respondents reported prior experience with IPTCs, indicating moderate familiarity with ideological and political education.

### 3.4. Reliability and validity testing

To ensure the robustness of the measurement instrument, the reliability and validity of the adapted Ideological and Political Theory Course Student Engagement Scale (IPTCSSE) were rigorously examined. The IPTCSSE has been widely applied in studies on ideological and political education in Chinese universities and is theoretically grounded in the tripartite engagement model (behavioral, cognitive, emotional) proposed by Fredricks et al. (2004).

#### 3.4.1. Expert validity testing.

Prior to data collection, expert review was conducted to evaluate the content validity and contextual appropriateness of the adapted scale. Three senior scholars in ideological and political education and two experts in educational psychology independently assessed each item for relevance, representativeness, and linguistic clarity. Based on their suggestions, minor modifications were made to item wording and translation to enhance comprehensibility. All experts agreed that the revised instrument adequately captured the key aspects of student engagement in ideological and political theory courses.

#### 3.4.2. Construct validity.

(1) Confirmatory Factor Analysis and Model Fit

Confirmatory factor analysis (CFA) was conducted to assess the construct validity of the IPTCSSE scale. The hypothesized three-factor model (behavioral, cognitive, emotional engagement) demonstrated satisfactory fit to the data (see Table 2).

**Table 2:** Confirmatory factor analysis and model fit.

Fit Index	Value	Recommended Threshold
χ²/df	2.48	<−3
CFI	0.93	≧0.90
TLI	0.91	≧0.90
RMSEA	0.056	<0.08
SRMR	0.045	<0.08

Construct validity was assessed through confirmatory factor analysis (CFA) using AMOS 26.0. The three-factor model demonstrated good fit to the data (χ²/df = 2.48, CFI = 0.93, TLI = 0.91, RMSEA = 0.056), confirming the hypothesized structure of behavioral, cognitive, and emotional engagement. The standardized factor loadings ranged from 0.71 to 0.89, all significant at the 0.001 level. The composite reliability (CR) values for the three subdimensions ranged between 0.86 and 0.91, and the average variance extracted (AVE) values were between 0.61 and 0.68. These values surpass the commonly accepted thresholds (CR ≥ 0.70; AVE ≥ 0.50), indicating satisfactory convergent validity.

#### 3.4.3. Reliability testing.

Internal consistency reliability was confirmed through Cronbach’ s α coefficients, which were 0.86 for behavioral engagement, 0.88 for cognitive engagement, and 0.91 for emotional engagement. The overall Cronbach’s α for the full scale was 0.89, reflecting excellent reliability.

#### 3.4.4. Saturation and coverage.

Because the questionnaire was adapted from an existing validated instrument, saturation testing—typically required for self-developed scales—was not necessary. Nevertheless, the inclusion of two open-ended reflection items enabled triangulation with qualitative data. The thematic analysis of these responses confirmed that the three dimensions effectively captured the full range of students’ behavioral, cognitive, and emotional engagement experiences, thereby ensuring theoretical and empirical completeness.

Taken together, these results demonstrate that the adapted IPTCSSE exhibits robust psychometric properties and provides a reliable and valid measure of student engagement within the ideological and political education context.

### 3.4. Data process

Emotional engagement is a key dimension of student engagement, reflecting students’ affective responses to learning experiences. Prior research has emphasized its multifaceted nature, including factors such as: Interest, which is the degree to which students are curious or intrigued by course content. Enjoyment, which is the pleasure or satisfaction derived from learning activities. Pride, which is positive feelings associated with learning achievements or alignment with course values. Emotional identification, which is the extent to which students feel connected to the curriculum or its ideological content. These factors are particularly relevant in ideological and political education, where emotional engagement can influence students’ motivation, attitudes, and acceptance of course principles. This study adopts a cross-sectional survey design. Although the section previously mentioned the potential value of longitudinal research, this study does not implement a longitudinal design. Instead, it analyzes emotional engagement at a single point in time. Longitudinal investigations are recommended for future studies. Overall, these results demonstrate that the adapted questionnaire possesses strong reliability and validity and effectively measures the full range of behavioral, cognitive, and emotional engagement among Chinese university students in ideological and political theory courses.

## 4. Results

To examine the factors influencing students’ emotional engagement in Ideological and Political Theory Courses (IPTCs), we conducted a linear regression analysis including four predictor dimensions: behavioral engagement, cognitive engagement, emotional engagement, and relevant course-related factors (e.g., prior exposure to IPTCs).

### 4.1. Factor analysis of emotional engagement in college IPTCs

#### 4.1.1. KMO and bartlett’s test of sphericity.

The KMO value for the emotional engagement subscale for college IPTCs was 0.706, which falls between 0.7 and 0.8. This suggests that the data are appropriate for factor analysis. Additionally, Bartlett’s sphericity test shown that Approximate chi-square is 117.073, Degrees of freedom is 91, Significanceis is.034 and yielded a significance probability of P < 0.05, indicating that the questionnaire had strong structural validity and was suitable for factor analysis.

#### 4.1.2. Extraction of common factors.

After performing several exploratory factor analyses on the emotional engagement subscale data for college ideological and political courses, we used principal component analysis to examine the total variance explained by each factor component of emotional engagement in these courses. The results are shown in Table 3.

**Table 3:** Total Variance explained by emotional engagement factors in IPTCs.

	Initial Eigenvalues			Extracted Sum of Squared Loadings		
Components	Total	Variance Percentage (%)	Cumulative (%)	Total	Variance Percentage (%)	Cumulative (%)
1	1.125	8.039	8.039	1.125	8.039	8.039
2	1.085	7.749	15.787	1.085	7.749	15.787
3	1.074	7.669	23.456	1.074	7.669	23.456
4	1.057	7.548	31.004	1.057	7.548	31.004
5	1.033	7.379	38.382	1.033	7.379	38.382
6	1.020	7.283	45.665	1.020	7.283	45.665
7	1.005	7.180	52.846	1.005	7.180	52.846
8	.974	6.956	59.801			
9	.969	6.922	66.723			
10	.961	6.865	73.588			
11	.950	6.782	80.371			
12	.939	6.711	87.082			
13	.907	6.481	93.563			
14	.901	6.437	100.000			

As shown in Table 3, by the time we reach the second component, 14 items account for 45.459% of the total variance. This analysis identifies 7 factors with eigenvalues greater than 1. According to the rotated component matrix, the first factor includes items 1 and 4; the second factor includes items 10 and 12; the third factor includes items 6 and 11; the fourth factor includes item 2; the fifth factor includes item 3; the sixth factor includes items 9 and 13; and the seventh factor includes items 7, 8, and 14.

The scree plot reveals that the eigenvalues for the first 7 factors are greater than 1 whereas those from the 8th factor onward are less than 1, indicating an inflection point. Consequently, we select the first 7 factors.

#### 4.1.3. Factor analysis table of college students’ emotional engagement.

Considering the characteristics of college students and integrating the literature with the questionnaire items, we conduct a comprehensive factor analysis of emotional engagement for college students. The identified factors are interest and belonging, peer relations, family and curriculum, emotional state, identity, teacher support and influence, and course satisfaction, as detailed in Table 4. Emotions reflect individuals’ internal experiences and attitudes toward objective phenomena, with positive emotions serving as a powerful motivator for learning activities among college students. In ideological and political theory courses, positive emotions are crucial and act as catalysts for educational success. They motivate students, enhance the effectiveness of these courses, and help students internalize the impact of educational activities. Understanding these key factors is essential for improving emotional engagement and strengthening the overall effectiveness of ideological and political courses.

**Table 4:** Factor analysis table of college students’ emotional engagement in ideological and political theory courses.

Items	Indicators	Factor naming						
		Interest Belonging	Classmates /Peers	Family Course	Emotions and Feelings	Sense of Identity	Teacher Support and Influence	Sense of Achievement in the Course
B1	Interest	0.561						
B2	Emotions				0.592			
B3	Sense of Cognition					0.511		
B4	Sense of Belonging	0.557						
B5	Sense of Identity							
B6	Family Education			0.441				
B7	Sense of Achievement in the Course							0.47
B8	Course Difficulty							0.401
B9	Teacher Influence						0.455	
B10	Peer Influence		0.555					
B11	Achievement Value			0.538				
B12	Peer Influence		0.435					
B13	Teacher Support						0.594	
B14	Course Utility							0.588

### 4.2. Descriptive statistics: college students’ emotional engagement in IPTCs

The table presents descriptive statistics of college students’ emotional involvement in ideological and political education.

As shown in Table 5, the maximum value for the interest and belonging factor is 10.00, the minimum value is 4.00, and the mean is 6.6508. For the influence of classmates and peers, the maximum value is 10.00, the minimum value is 4.00, and the mean is 6.2282. Similarly, for the impact of family and course, the maximum value is 10.00, the minimum value is 4.00, and the mean is 6.3988. Emotional factors have a maximum value of 10.00, a minimum value of 4.00, and a mean of 6.6979. The sense of identification has a maximum value of 10.00, a minimum value of 4.00, and a mean of 6.4671. Teacher assistance and influence have a maximum value of 10.00, a minimum value of 4.00, and a mean of 6.3551. The sense of course achievement has a maximum value of 14.50, a minimum value of 6.00, and a mean of 9.5606.

**Table 5:** Descriptive statistics of college students’ emotional involvement in IPTCs.

	N	Minimum value	Maximum value	Mean	Standard deviation
Interest and Belonging	3992	4.00	10.00	6.6508	1.09608
Classmates and Peers	3992	4.00	10.00	6.2282	1.19755
Family and Curriculum	3992	4.00	10.00	6.3988	1.19352
Emotions and Feelings Sense of Identity	3992 3992	4.00 4.00	10.00 10.00	6.6979 6.4671	1.19996 1.19471
Teacher Assistance and Influence	3992	4.00	10.00	6.3551	1.21925
Sense of Achievement in Courses	3992	6.00	14.50	9.5606	1.56452
Effective Case Count (in columns)	3992				

As shown in Table 5, the standard deviations are as follows: 1.09608 for interest and belonging, 1.19755 for classmates and peers, 1.19352 for family and course, 1.19996 for emotional factors, 1.19471 for sense of identification, 1.21925 for teacher assistance and influence, and 1.56452 for a sense of course achievement. These relatively stable standard deviation scores indicate low variability, suggesting that these factors consistently influence college students’ emotional engagement in ideological and political education.

Additionally, the dataset shows no outliers because all the means fall within the range of the minimum and maximum values. The mean scores for interest and belonging, classmates and peers, family and course, emotional factors, sense of identification, teacher assistance and influence, and sense of course achievement are all above 6, indicating an overall positive evaluation. Among these factors, the sense of course achievement has the highest mean score (M = 9.5606), whereas the influence of classmates and peers has the lowest mean score (M = 6.2282). This suggests that the sense of achievement in the course is the most significant factor that influences college students’ emotional engagement in ideological and political education, whereas the impact on classmates and peers is relatively weaker.

### 4.3. Correlation and linear regression analysis of college students’ emotional engagement in IPTCs

Before conducting regression analysis, it is essential to perform correlation analysis. Correlation analysis allows researchers to preliminarily assess whether there are relationships among the data.

#### 4.3.1. Correlation analysis.

Table 6 shows that college students’ emotional engagement in ideological and political education is significantly positively correlated with other variables, including interest and belonging, classmates and peers, family and course, emotional factors, a sense of identification, teacher assistance and influence, and a sense of course achievement.

**Table 6:** Correlation Analysis of College Students’ Emotional Engagement in IPTCs.

			Interest and Belonging	Classmates and Peers	Family and Course	Emotions and Feelings	Sense of Identification	Teacher Assistance and Influence	Sense of Course Achievement
Emotional Engagement	Pearson correlation	1	.303^**^	.361^**^	.365^**^	.377^**^	.346^**^	.352^**^	.474^**^
	Sig.(two tails)		.000	.000	.000	.000	.000	.000	.000

Note: *p < 0.05; **p < 0.01; ***p < 0.001.

#### 4.3.2. Linear regression analysis.

As shown in Table 7, R2 = 0.73, F = 1834.01, P = 0.000, indicating that interest and belonging, classmates and peers, family and course, emotional factors, a sense of identification, teacher assistance and influence, and a sense of course achievement are all positively correlated with college students’ emotional engagement in ideological and political education. Among these factors, interest and belonging have the highest weights (eigenvalue = 0.04), whereas the sense of course achievement has the lowest weight (eigenvalue = 0.01).

**Table 7:** Linear regression analysis of college students’emotional engagement in IPTCs.

Model		Unstandardized Coefficients		Standardized Coefficients			Collinearity Statistics		R^2^	F
		B	Standard Error	Beta	t	Significance	Eigenvalue	VIF		
1	(Constant)	0.87	0.05		16.28	0	6.84			1834.01
	Interest and Belonging	0.14	0.00	0.31	38.41	0	0.04	1.00		
	Classmates and Peers	0.10	0.00	0.23	25.25	0	0.03	1.29		
	Family and Course	0.15	0.00	0.36	44.23	0	0.03	1.00	0.73	
	Emotions and Feelings	0.14	0.00	0.34	41.77	0	0.03	1.00		
	Sense of Identification	0.13	0.00	0.33	39.27	0	0.02	1.04		
	Teacher Assistance and Influence	0.11	0.00	0.26	28.12	0	0.02	1.29		
	Sense of Course Achievement	0.15	0.00	0.47	57.31	0	0.01	1.00		

## 5. Discussion

### 5.1. Principal factors

#### 5.1.1. Interest and belonging.

Interest represents an individual’s cognitive inclination toward various subjects and reflects their emotional engagement. The emergence of interest prompts individuals to explore new knowledge and develop new skills. In the realm of ideological and political education, interest plays a crucial role and is a key focus for researchers. It serves as a central psychological factor that influences learning behavior.

Interest in ideological and political education typically comprises three critical elements: attitude, need, and emotion. To cultivate college students’ interest in these subjects, attention must be given to factors such as the educational environment, content, and teaching methods. College students often exhibit a lack of enthusiasm for ideological and political courses, which manifests in insufficient recognition of the significance of these courses and results in passive learning behaviors and utilitarian motivations.

When college students possess strong situational interest, they actively engage with the content, assess their learning capabilities, respond to changes in the learning environment, and maintain positive expectations for learning outcomes. In such an environment, when students can connect their learning content with their interests and understanding, their motivation to learn increases significantly. This leads to more successful learning outcomes.

Ideological and political education in college aims to equip students with the ability to apply Marxist positions, viewpoints, and methods flexibly to allow students to understand, analyze, and address problems in depth and to consciously transform their subjective world to enhance their ideological and moral development. Ideological and political courses serve as the primary channels for this education. Compared with other subjects, these courses are more political and theoretical in nature. Enhancing college students’ interest in these courses through effective teaching methods is crucial for achieving the goals of ideological and political education. It is essential to choose appropriate teaching strategies that stimulate students’interest, improve educational effectiveness, and foster a strong sense of belonging to promote their overall growth.

#### 5.1.2. Classmates and peers.

The influence of classmates and peers is crucial for the emotional engagement of college students in ideological and political theory courses. In practice, some students have relatively weaker learning abilities and improper attitudes toward these courses, which poses new challenges for improving the learning atmosphere, increasing the efficiency of education on ideological and political theory, and creating a healthy and positive learning environment. Peer influence can help address this educational reality because peer learning, mutual support, and solidarity among students may be more direct and effective than direct instruction and repeated advice from teachers. By applying new methods and concepts in ideological and political education, peer education among students can establish stable and flexible peer influence. This approach leverages both collective education and individual influence to create new forms and methods for enhancing emotional engagement in ideological and political theory courses.

#### 5.1.3. Family and curriculum.

Generally, the environment for ideological and political education can be categorized into the microenvironment and the macroenvironment. The microenvironment includes family, school, and work settings, whereas the macroenvironment includes political, economic, and cultural contexts. The family, in particular, is a crucial venue for ideological and political education and serves as a foundational influence on the thoughts and values of its members. This process is ongoing and often benefits from its emotional impact.

In ideological and political education, the unique role of traditional family culture is highly significant. Both material and spiritual aspects of the family environment impact ideological and political courses. Factors such as the family’s economic status, social relationships, family structure, and dynamics among family members inevitably influence college students’ engagement with these courses. The educational values and attitudes present within the family subtly shape students’ ideological behavior.

Ideological and political theory courses at universities offer a comprehensive educational experience for college students. This experience can be both explicit and implicit but is always deliberate and purposeful and covers a range of educational content and factors. The most explicit element of IPTCs is the textbook, which constitutes the core of these courses and serves as the primary vehicle. Other educational activities aimed at enhancing students’ ideological and moral character, though often less direct, are also integral to the ideological and political education framework. Moreover, as IPTCs have become increasingly integrated into the curriculum, their alignment with broader curriculum ideology has deepened across diverse colleges and universities. This evolving integration is giving rise to new educational approaches and shaping a fresh landscape for ideological and political education.

#### 5.1.4. Emotions and feelings.

Emotional factors significantly impact modern ideological and political theories. These factors affect both the cognitive development and the psychological processes of college students and foster a more harmonious relationship between educators and learners. Emotions can influence both teaching and learning in these courses. To enhance engagement and passion for ideological and political theory, it is crucial to acknowledge the role of emotions for both educators and students, offer correct guidance, and incorporate innovative content into teaching methods. Providing students with positive emotional experiences in these classes through appropriate methods helps to establish a stable emotional state and enhances the effectiveness of ideological and political education.

#### 5.1.5. Sense of identification.

Through classroom ideological and political education, college students’ identification with these subjects can be strengthened, thereby increasing their effectiveness. College students are not only the hope and the future of the nation but also key contributors to socialist development. Their ideological and moral qualities are closely linked to the country’s long-term progress, and their sense of national identity is vital for the development of the Chinese nation.

Recently, college students have been actively and healthily developing their ideological and political awareness, as demonstrated by their ongoing engagement with major international and domestic events and their increasingly mature and rational value judgments. With the acceleration of economic globalization and the trend toward multipolarity in international politics, China’s economic structure has become more diverse. This has resulted in a broader and more complex range of ideological views and value orientations among university students, which poses greater challenges for ideological and political education in China.

In IPTCs, increasing emotional engagement can encourage college students to embrace greater responsibilities toward their nation, develop stronger identification with socialist economic and political systems, gain a clearer understanding of their roles, and foster a deeper desire to contribute to their country’s prosperity and strength. This sense of identification with the nation, family, and self is a crucial component of students’ emotional involvement in ideological and political education.

#### 5.1.6. Teacher support and influence.

The ideological and political education of teachers is not only a primary aspect of organizing and implementing these classes but also the key force in executing educational work and significantly influences its effectiveness. The task of nurturing university students and enhancing their emotional involvement in IPTCs is closely tied to the role of these teachers.

Various factors, including a teacher’s subject awareness, autonomy, emotional state, reasonable material needs, and personal interests, influence the work of teachers of ideological and political education. The essence of ideological and political education is reflected in a form of humanistic care, particularly with regard to the material, spiritual, and social needs of these teachers based on their real-life conditions and requirements. This aspect should be emphasized.

This humanistic care is conveyed to college students through ideological and political education teachers, who influence students and enhance their engagement with the subject. This leads to improved professional skills, purer thoughts, and more comprehensive personal development. Therefore, it is crucial to research students’emotional involvement in ideological and political classes, support the development of university teachers in this field, enhance their personal qualities, update their teaching methods, and establish a student-centered teacher‒student relationship. Teachers of ideological and political education should continually encourage college students to take initiative in their learning, promote inquiry-based learning methods, and foster a lifelong commitment to education. They should also help students expand their knowledge base and develop strong educational and research skills.

#### 5.1.7. Sense of achievement in courses.

In recent years, achieving a sense of accomplishment in ideological and political education classes has become a key goal in universities. This pursuit serves as an intrinsic value, a driving force for reform, and a criterion for evaluation. Through these classes, college students gain valuable knowledge and benefits and fulfill their fundamental needs for growth and development. This is because ideological and political classes provide college students with insights into ideological and moral understanding, political awareness, value alignment, and spiritual support. Positive and meaningful experiences in these classes encourage students to invest more deeply in ideological and political education. This sense of achievement is essential for helping students discern right from wrong, distinguish good from evil, and align with their ideals and beliefs as well as their commitment to the great cause of the Chinese nation.

Enhancing college students’ emotional engagement in ideological and political classes involves continuously improving the appeal and relevance of these courses to meet students’ needs and expectations during their growth and development. In other words, when students find classes effective and engaging, they are more likely to invest their genuine feelings and emotions, thereby increasing their sense of achievement. Therefore, it is essential to strengthen the integration of core socialist values into ideological and political education, address the developmental needs of college students, and fully leverage the comprehensive educational potential of these classes.

### 5.2. Analysis

#### 5.2.1. The high score of the sense of course achievement.

These findings indicate that college students experience consistently positive subjective feelings throughout the process of ideological and political education. These feelings include psychological, intellectual, and behavioral aspects and arise from a combination of various factors and the deep integration of internal and external influences. They represent the spiritual growth and insights students gain through their engagement with ideological and political education.

This sense, which is derived from personal experience and felt deeply in the spirit, reflects individuals’ psychological expectations. Through dedicated and attentive teaching in ideological and political classes, educators aim to cultivate greater emotional engagement among students, thereby enhancing their overall competence and sense of responsibility toward their country and nation. During their university years, students are in the process of shaping their values and are challenged in their ability to discern right from wrong and navigate their future. Ideological and political education provides them with a window into understanding the world and offers insights into both international and domestic situations. This exposure helps students gain a clearer understanding of China’s unique national conditions and the policies and guidelines of the party and government. As a result, they develop a deeper awareness of social ideals, a commitment to the great cause of socialism with Chinese characteristics, and overall reflection on the effectiveness of ideological and political education.

#### 5.2.2. Low scores for classmates and peers.

Within the context of ideological and political education, the influence of classmates and peer groups, which is an important factor in the microenvironment, may have a reduced impact on college students’ ideological and political literacy. With advances in information technology, the role of classmates and peers in shaping students’ emotional engagement in ideological and political classes may no longer be as significant as it once was.

On the one hand, because undergraduates are already adults, their values are less influenced by classmates and peers. Individuality becomes more pronounced, and while group characteristics remain, peer influence gradually diminishes. Most students develop the ability to independently assess and form their own preferences and opinions. Consequently, opportunities for engaging with information may surpass opportunities for peer interactions. On the other hand, the emotional engagement component of ideological and political theory courses may not be sufficiently stimulated. In the current context of ideological and political education, college students often overlook emotionally supportive campus resources, positive peer relationships, healthy emotional management, and empathetic teaching environments. As the British scholar Diana Coyle noted, the world is increasingly becoming a “weightless world”. Some students may avoid real-world problems and issues, preferring the more abstract realm of the internet and engaging more with digital texts than with their classmates and peers. In doing so, they may sacrifice genuine emotions and sincerity for the transient while neglecting the tangible realities of their immediate surroundings.

#### 5.2.3. Emotional engagement slightly higher than behavioral and cognitive engagement.

The present study found that students exhibited moderately high emotional engagement in Ideological and Political Theory Courses (IPTCs), with emotional engagement slightly higher than behavioral and cognitive engagement. These findings are consistent with prior research indicating that affective responses play a critical role in shaping students’ participation and learning outcomes in ideological education (Fredricks et al., 2004; Pekrun, 2006). For example, students’ emotional engagement significantly predicts their motivation and attitude toward political and civic education, which aligns with our observation that emotional engagement was a key dimension in IPTCs. Moreover, the observed positive correlations between behavioral, cognitive, and emotional engagement also mirror findings from Kahu (2013) and Reeve and Tseng (2011), who highlighted the interdependent nature of engagement dimensions. Our results extend this literature by demonstrating that, within the context of Chinese IPTCs, curriculum-related factors further influence emotional engagement, reinforcing the notion that instructional design and content relevance are critical for fostering affective engagement.

Some differences from prior studies were also noted. While earlier research often reported higher behavioral engagement than emotional engagement, our study found the opposite pattern. This discrepancy may be attributed to recent pedagogical reforms in Chinese universities, increased emphasis on interactive teaching methods, and curriculum content that more effectively resonates with students’ values and interests. Overall, by comparing our results with prior research, we can conclude that the multidimensional model of student engagement is applicable to IPTCs, and that emotional engagement serves as a central factor linking curriculum design to students’ attitudes and learning experiences. These comparisons highlight the theoretical and practical implications for curriculum development, instructional strategies, and future research on student engagement in political education.

### 5.3. Descriptive statistics

In the descriptive statistics section, the mean and standard deviation for each factor of emotional engagement (interest, enjoyment, pride, and emotional identification) are presented. This consistency across the literature review, scale description, and results provides a clear conceptual framework for interpreting students’ affective engagement in IPTCs. By explicitly connecting our results with the studies reviewed in the literature, we provide a coherent narrative that situates our findings within the broader body of research and clarifies the study’s contribution to understanding student engagement in ideological and political education.

## 6. Conclusion

Based on the analysis, we provide actionable suggestions for educators and policymakers to increase the effectiveness of ideological and political theory courses. Peer support is a critical predictor of college students’ emotional engagement and affects both their level of emotional involvement and their degree of detachment. Peer influence is a significant factor that contributes to the variation in emotional engagement experienced by students.

### 6.1. Suggestions

The study findings directly address the stated objectives. First, emotional engagement was identified as a central component of student participation in IPTCs, confirming the relevance of measuring behavioral, cognitive, and emotional dimensions. Second, curriculum-related factors were shown to influence students’ attitudes and feelings, highlighting the role of instructional design in fostering engagement. These conclusions demonstrate a clear connection between the study objectives, research methods, and results, providing both theoretical insights and practical recommendations for improving student engagement in ideological and political education.Finally, the classroom is a setting where effective emotions can be fostered through peer interaction, thereby enhancing engagement. Modern educational philosophy views peer interaction as crucial and beneficial. In ideological and political classrooms, students—acting as a group of ‘peers’—learn together to achieve common goals. They participate in group activities and remain relatively independent yet integral to the collective effort, which helps to create a unified and positive learning atmosphere. In this environment, students do not merely absorb knowledge passively but also engage in interactive discussions to evaluate information and distinguish truth from falsehood. Although this approach may challenge the teacher’s authority, it fosters students’ autonomy in learning. In ideological and political classrooms, peer interaction should focus on specific learning tasks and avoid being aimless or casual. Students should fully engage in this interactive process, making it a structured and effective means of emotional involvement.

This study provides insights into the emotional engagement of students in Ideological and Political Theory Courses (IPTCs) and identifies factors influencing their affective responses. The findings underscore the importance of considering behavioral, cognitive, and emotional dimensions of engagement, as well as curriculum-related influences, to foster positive student experiences in political education.

### 6.2. Limitations

Despite its contributions, the study has several limitations. First, the data were collected via self-reported questionnaires, which may be affected by social desirability or recall bias. Second, the sample was drawn from only some universities, which may limit the generalizability of the findings to other institutions or regions. Third, the cross-sectional design prevents causal inference regarding the relationships between engagement dimensions and curriculum factors.Finally, a key limitation of this study is its cross-sectional nature. Without longitudinal data, we cannot assess how students’ emotional engagement evolves across semesters or how IPTCs exert long-term influence on students’ ideological understanding and value formation.

### 6.3. Future research directions

Future studies could employ longitudinal designs to examine changes in student engagement over time and incorporate observational or experimental methods to strengthen causal conclusions. Expanding the sample to include diverse institutions would enhance generalizability. Furthermore, research could investigate additional contextual factors, such as teacher-student interactions, pedagogical innovations, or curriculum content variations, to provide a more comprehensive understanding of factors influencing emotional engagement in IPTCs.

By acknowledging these limitations and proposing avenues for future research, this study provides a foundation for ongoing investigation into enhancing student engagement and the effectiveness of ideological and political education in higher education. IPTC instructors should incorporate interactive and participatory teaching methods, such as group discussions, debates, and case studies, to foster behavioral, cognitive, and emotional engagement. Teachers can integrate content that resonates with students’ values and experiences, such as real-life examples, historical case studies, or narratives of role models, to enhance affective engagement. Regular feedback mechanisms and opportunities for student reflection can help monitor engagement levels and adjust teaching strategies in real time.
